# Promoter Frame Position Affects Strength and Nature of Circadian Oscillations in *hPER2* Luciferase Reporters

**DOI:** 10.3390/ijms262110785

**Published:** 2025-11-06

**Authors:** Bhavna Kalyanaraman, Gabrielle Villafana, Stephanie R. Taylor, Michelle E. Farkas

**Affiliations:** 1 Department of Chemistry, University of Massachusetts Amherst, Amherst, MA 01003, USA; 2 Molecular and Cellular Biology Graduate Program, University of Massachusetts Amherst, Amherst, MA 01003, USA; 3 Department of Computer Science, Colby College, Waterville, ME 04901, USA; srtaylor@colby.edu

**Keywords:** circadian rhythms, *PERIOD 2*, promoter-reporter, firefly luciferase, lentiviral expression, U2OS cells, rhythmicity

## Abstract

The *PER2* gene is a crucial component responsible for the proper functioning of the mammalian core circadian clock. The circadian nature of the murine *Per2* (*mPer2*) promoter’s activity has been thoroughly investigated to identify important elements responsible for its oscillatory behavior; however, its human counterpart has not. While there are similarities between murine and human core clocks, there are differences and unconserved elements between their promoter sequences that may influence the nature of rhythms. Further, most studies to date have used murine-based sequences in human cell lines. To fully understand the role(s) of and factors involved in the human *PER2* (*hPER2*) gene, human-derived sequences should be used. To this end, we developed two lentiviral luciferase reporters in well-established, circadian model U2OS cells using different *hPER2* promoter regions. Their rhythmic nature was compared to that of the standard *mPer2* promoter reporter. We found that *hPER2* reporters exhibited stronger oscillations than the *mPer2* reporter, and that the frame of the *hPER2* promoter affected the period and phase. This work introduces a human sequence-based *PER2* promoter in U2OS cells, which should be used for further in vitro tracking of *hPER2* activity and to understand *PER2* gene dynamics, in lieu of the murine iteration.

## 1. Introduction

Circadian rhythms are innate biological processes that regulate various functions in an organism. They follow an approximately 24 h cycle that is driven by the master clock, located in the suprachiasmatic nucleus (SCN) [1,2]. The SCN receives external environmental cues, primarily light, to entrain itself, as well as peripheral oscillators distributed throughout the body [2,3]. The widely accepted molecular model of the core clock is composed of a cell-autonomous, auto-regulatory transcriptional-translational feedback loop (TTFL) [4]. The positive arm of the TTFL includes brain and muscle ARNT-like-1 (BMAL1), and circadian locomotor output cycles kaput (CLOCK), which heterodimerize and drive the expression of various clock-controlled genes, including periods (PER1/2/3) and cryptochromes (CRY1/2). Upon reaching threshold concentrations, PER and CRY form an inhibitory complex that interacts with CLOCK:BMAL1, resulting in the negative regulation of the TTFL. The use of various biochemical assays, including protein pulldown assays [5,6], chromatin immunoprecipitation (ChIP) [5,7], and RT-PCR [5,6,7,8], has been important in providing us with information about the key aspects of the negative arm of the TTFL. Reporters have also played a crucial role in monitoring the real-time dynamics of core clock components. The generation of promoter-based reporters, as well as fusion proteins expressing luminescent or fluorescent reporters, has facilitated the monitoring of circadian dynamics at both transcriptional and translational levels [9,10,11,12,13,14].

Seminal work by Yoo et al. introduced PER2::luciferase (Per2::luc) mice, which allowed tracking of PER2 protein oscillations in vivo [9]. This study highlighted the importance of PER2 in producing self-sustained oscillations in peripheral tissues. To date, Per2::luc mice and tissues isolated from this reporter model have been widely used, expanding our knowledge of the circadian clock [12,15,16,17,18]. Some of these studies have shown that the period and phase of the molecular clock are controlled by PER2, thereby making it a stoichiometrically rate-limiting component of the primary TTFL [19,20]. To understand the transcriptional regulation of the *Per2* promoter, several iterations of mouse *Per2* (*mPer2*) promoter sequences with varying lengths have been used to develop *mPer2:luc* promoter reporters [21,22]. These studies have revealed the presence of an E’-box (5′-CACGTT-3′), which is essential for retaining the rhythmicity of the *Per2* promoter. Additionally, it was established that using a truncated minimal proximal *mPer2* promoter of approximately 200 base pairs (bp) around the transcript start site (TSS) (containing the E2 enhancer) was sufficient to drive robust rhythms for *Per2* in peripheral cells. These studies also showed that introducing mutations to the E2-enhancer resulted in the loss of rhythms.

The transcriptional-level circadian nature of *Per2* has been extensively studied and monitored for various applications in vitro, using reporters expressing truncated murine *Per2* promoters in NIH3T3 (mouse fibroblast) [23,24] and U2OS (human bone osteosarcoma) cells [25,26], among others [10,27,28]. To date, it has been standard practice to use mouse sequence-derived circadian reporters in human cell lines in vitro, particularly U2OS [10,25,26,28,29,30,31], which serves as a standard in vitro model to study circadian rhythms [29]. This practice is supported by the presence of conserved regions, including the E’-box, hypoxia response element (HRE), and the *mPer2* transcription start site (+1), which is shared by both the *mPer2* and *hPER2* promoters. However, it is important to also recognize that there are differences between the murine and human core clock in terms of genomic regulation. A recent transcriptomic study revealed differences in the rhythmicity of various clock-controlled transcripts in human and mouse prefrontal cortex (PFC) samples [32]. This could arise from cross-species variations in the unconserved regions, which may alter the circadian nature of the gene and, in turn, could introduce deviations in comparing the nature of oscillations of *Per2* at the promoter level to those of the translated PER2 protein. In addition to the variations in the genomic sequence, the co-evolution of transcription factors across species [33] might also have an effect on the nature of the promoter activity. In fact, previous in vitro studies reported discrepancies between assessments of murine promoter activity and endogenous protein expression levels in human cells [10,27,34,35]. There have been a few studies that have used the *hPER2* promoter sequences to evaluate circadian effects due to mutations in E-boxes [35,36] and secondary circadian regulators [37]. However, these studies did not assess the rhythmic activity of the *hPER2* promoter. Therefore, our knowledge of the transcriptional regulation of *hPER2* is limited.

Here, we sought to bridge this gap in knowledge by using lentiviral luciferase reporters bearing shifted frames of the *hPER2* promoter fragment, *hPER2.1:luc* and *hPER2.2:luc*. These reporters were incorporated into U2OS cells, and their rhythmic nature and characteristics were evaluated and compared to those derived from *mPer2:luc* promoter-reporters. We observed that the *hPER2* reporters exhibited strong oscillations with amplitudes higher than those of *mPer2* reporters. Additionally, our findings revealed that the upstream shifted *hPER2* promoter-reporter (*hPER2.1:luc*) resulted in a higher period and phase delay, while the downstream shifted promoter-reporter (*hPER2.2:luc*) exhibited a period and phase similar to the previously established *mPer2:luc* promoter-reporter. Taken together, our study introduces human-derived *PER2:luc* promoter-reporters and demonstrates that shifting the frame of the *hPER2* promoter affects the nature of *PER2* oscillations in vitro. These reporter systems should be used to monitor the rhythmic activity of *PER2* transcription in human-derived in vitro models in the future, in order to provide a better understanding of its dynamics in health and disease, as well as its responses to perturbation.

## 2. Results and Discussion

### 2.1. Generation and Validation of hPER2 Promoter Reporter Constructs

**Figure 1:**
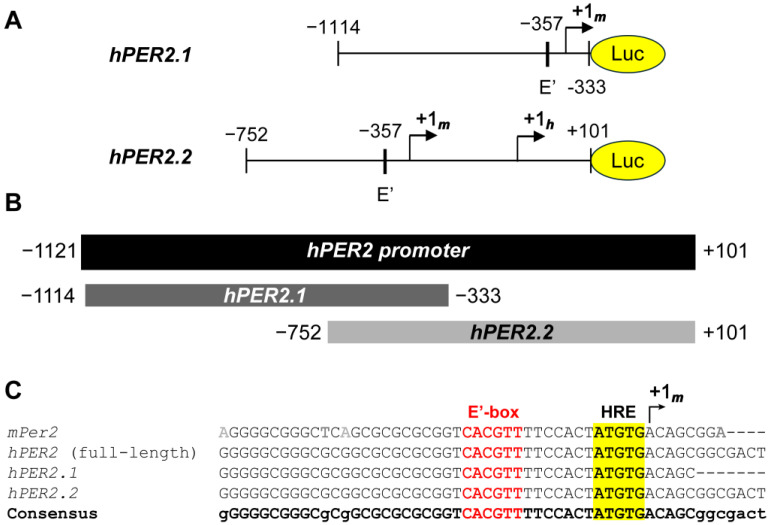
Analysis of *hPER2* promoter fragments. (**A**) A 781 bp truncated promoter (*hPER2.1*) and an 853 bp truncated promoter (*hPER2.2*) were sub-cloned upstream of the luciferase gene. (**B**) Graphical representation of the alignments of the two truncated *hPER2* promoter sequences against the full-length *hPER2* promoter sequence. (**C**) Sequence alignments of the 34 bp conserved regions for *hPER2.1* and *hPER2.2* promoter sequences against the full-length *hPER2* promoter sequence and the *mPer2* promoter sequence. The conserved E’-box (also known as the E2 enhancer; text in red) and the hypoxia response element (HRE; highlighted in yellow) are indicated in the sequences.

We obtained two truncated sequences of the *hPER2* promoter [38,39], and generated lentiviral luciferase promoter reporters using each, resulting in *hPER2.1:luc* and *hPER2.2:luc*, respectively (Figure 1A). To compare the frames of the two promoter fragments, we performed sequence alignment of the two promoter sequences against the full-length *hPER2* promoter, using the T-Coffee multiple sequence alignment (MSA) program [40]. We identified the full-length *hPer2* promoter by comparing the genomic sequence and the mRNA transcript sequence for the *hPER2* gene. We found the +1_h_ site by analyzing the functional and regulatory elements in the *hPER2* genomic sequence (Figure S1), which aligned with previous literature [21,41]. The *hPER2.1* promoter sequence aligned with the upstream region (−1114 to −333, relative to the *hPER2* transcription start site, +1_h_), while the *hPER2.2* fragment aligned with the downstream region (−752 to +101, relative to +1_h_) of the full-length *hPER2* promoter (Figure 1B). There was a 419-base pair (bp) region of overlap between *hPER2.1* and *hPER2.2* sequences, which contained E’-box (E2 enhancer; 5′-CACGTT-3′) and hypoxia response element (HRE) sequences and aligned with the consensus sequence. Further alignment with the truncated *mPer2* promoter from a previously established *mPer2:luc* promoter reporter [10,21] revealed a 34 bp conserved region with it, the E’-box, HRE, and the *mPer2* transcription start site (TSS), +1_m_ (Figure 1C). We identified the conserved +1_m_ in both *hPER2.1* and *hPER2.2* sequences. In addition to the +1_m_, the *hPER2.2* sequence also contained the +1_h_ site [41]. We also found a non-canonical E-box (5′-CAGGTG-3′; five bases upstream of +1_h_) in *hPER2.2*, which closely resembled the “minimal proximal promoter” organization described previously for *mPer2* [21]. Additionally, there were significantly longer frames of unconserved regions in both of the *hPER2* promoter sequences, which could contain regulatory elements unique to *hPER2*. Both *hPER2.1:luc* and *hPER2.2:luc* promoter reporter constructs were validated, by Sanger sequencing and whole plasmid sequencing, respectively.

**Figure 2:**
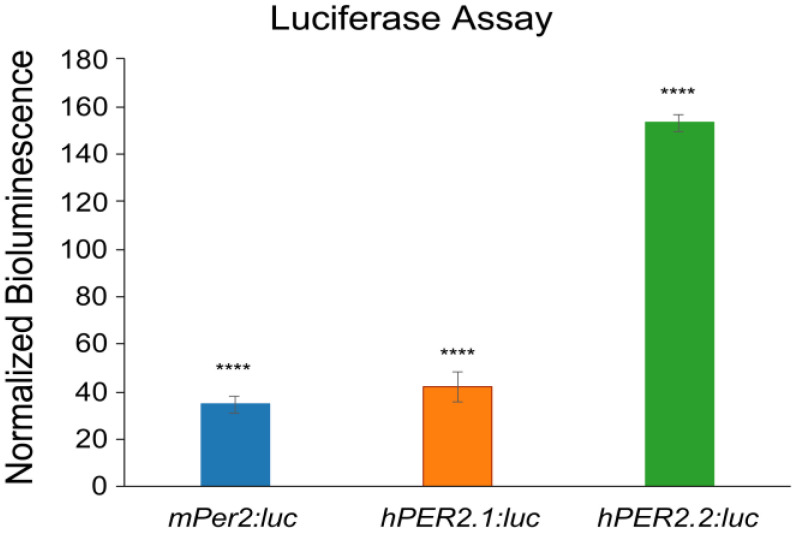
*PER2:luc* promoter reporter validation using luciferase assay. U2OS-*mPer2:luc*, U2OS-*hPER2.1:luc*, and U2OS-*hPER2.2:luc* cells were assessed for bioluminescence against the non-transfected (control) U2OS cell line using a luciferase assay. The data shown for each condition is an average of three replicates (N = 3), normalized to the control U2OS cells. Error bars represent standard deviation of the mean. A *t*-test was performed to compare the average bioluminescence intensities of the biological replicates for each cell line versus the control. (**** *p* < 0.0001).

Following validations, we stably transfected the *hPER2.1:luc* and *hPER2.2:luc* reporter constructs into U2OS (human bone osteosarcoma) cells using lentiviral transductions. After selection of the positively transfected cells, we performed a luciferase assay to validate the generation of a functional luciferase (Figure 2). We compared the bioluminescence intensities of U2OS-*hPER2.1:luc*, U2OS-*hPER2.2:luc*, and a U2OS-*mPer2:luc* cell line previously generated in our lab [26]. We observed that the normalized bioluminescence intensities of U2OS-*mPer2:luc*, U2OS-*hPER2.1:luc*, and U2OS-*hPER2.2:luc* were approximately 35-, 42-, and 153-fold, respectively, higher than the non-transfected U2OS control. The promoter activities of *mPer2:luc* and *hPER2.1:luc* have been established previously [26,38]. To discern the promoter activity of the newly established *hPER2.2:luc* promoter-reporter from baseline, we compared the luminescence signal from *hPER2.2:luc* against the same lentiviral vector backbone containing a non-specific (scrambled) insert upstream of the luciferase gene. A luciferase assay in U2OS cells transiently transfected with the two vectors revealed a significantly higher bioluminescence intensity in the U2OS-*hPER2.2* reporter cells, indicative of efficient *hPER2.2* promoter activity (Figure S2).

### 2.2. Assessment of hPER2 Promoter Frame Effects on Oscillations and Comparisons to mPer2 Traces

**Figure 3:**
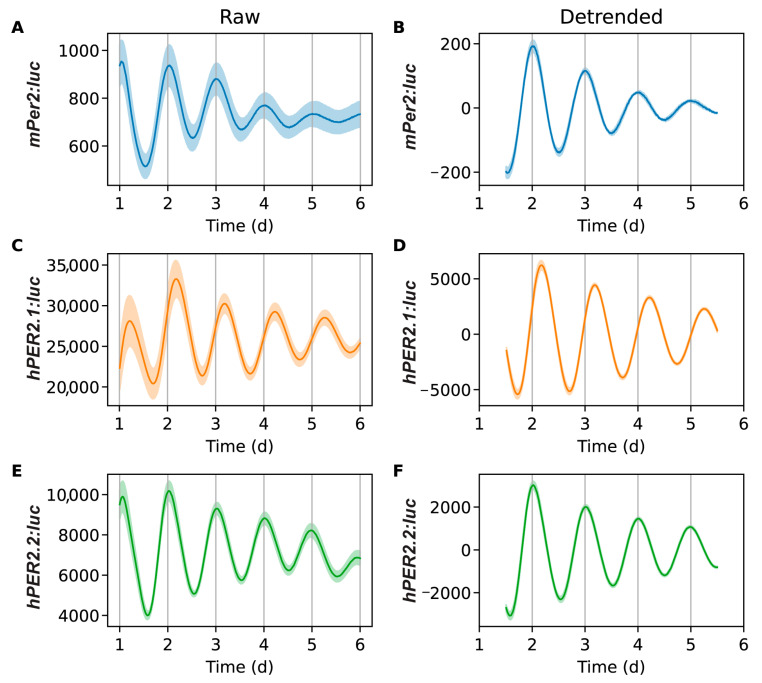
Bioluminescence time-series for *mPer2:luc* (**A**,**B**), *hPER2.1:luc* (**C**,**D**), and *hPER2.2:luc* (**E**,**F**). Excluding a 24 h transient, shown are raw time-series (**A**,**C**,**E**) and time-series after detrending (**B**,**D**,**F**) by subtracting the average of a 24 h moving window. The mean (raw or detrended) time-series is plotted as a solid line, with the standard error of the mean as a semi-transparent envelope around it. (N = 17 for *mPer2:luc* from three independent experiments, N = 24 for *hPER2.1:* from three independent experiments, and N = 16 for *hPER2.2:luc* from four independent experiments).

We performed luminometry assays to monitor the circadian behavior of our *hPER2* promoter-reporters and compare the nature(s) of their oscillations against the *mPer2* promoter reporter. We concurrently synchronized U2OS-*mPer2:luc*, U2OS-*hPER2.1:luc*, and U2OS-*hPER2.2:luc* cells for 2 h using a dexamethasone pulse, a synchronization method widely used for U2OS cells [25,26,31,42]. We recorded the bioluminescence signals for each cell line for seven days. The raw data was pre-processed by removing the first 24 h to eliminate the signal from transient peak expression. The data were detrended by subtracting a 24 h window moving average (Figure 3 and Figures S3–S5). The detrended data was then fit to a damped cosine curve.

The average bioluminescence intensity and oscillations of *mPer2* (Figure 3A,B) were consistent with our previously reported results [25,26,31]. Both *hPER2.1* and *hPER2.2* also exhibited robust oscillations (Figure 3C–F). We noted minor differences in the signals observed across different experiments (see Figures S3–S5); however, they remained largely consistent within each experiment. Our results showed that the amplitudes of the oscillations were stronger in both *hPER2.1* and *hPER2.2* when compared with the *mPer2* promoter reporter. This could be attributed to the use of longer lengths of the *hPER2* promoters (781 bp for *hPER2.1* and 853 bp for *hPER2.2*) compared to ~200 bp of the *mPer2*. While previous reports suggest that the use of the minimal *mPer2* promoter with the E’-box is sufficient to elicit rhythmic oscillations [21], there may be additional elements in the promoter that might be responsible for regulating the strengths and amplitudes of oscillations. Another reason for the higher amplitude of the *hPER2.1* promoter could be the presence of a miniCMV promoter downstream of the *hPER2.1* promoter. Furthermore, we used lentiviral transduction to generate stable reporter cell lines; however, we cannot control the number of promoter-reporter copies transfected per cell, which could be a contributing factor resulting in higher amplitudes. Off-target insertion of the reporter sequence into the genome is another pitfall of lentiviral transduction and could result in disruptions to normal cellular processes. We note that the cells did not have any detrimental changes to their physical (i.e., changes in cell morphology or cell division, vacuole indicative of cell stress, and cell death), or circadian attributes.

**Figure 4:**
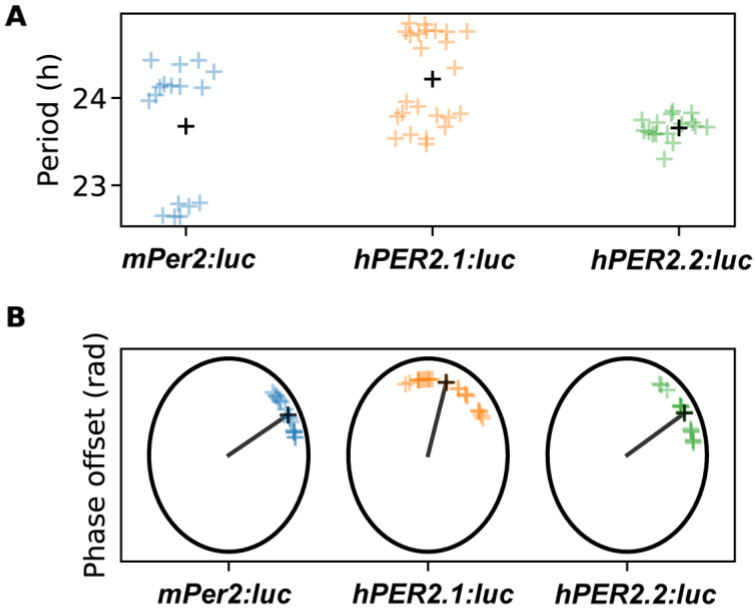
Shown are the period (**A**) and phase-offset (**B**) values estimated by fitting a damped cosine curve to de-trended *mPer2:luc*, *hPER2.1:luc*, and *hPER2.2:luc* time-series. The black plus (+) signs in (**A**) indicate average period, and the black line and x’s indicate average phase in (**B**). (N = 17 for *mPer2:luc* from three independent experiments, N = 24 for *hPER2.1:luc* from three independent experiments, and N = 16 for *hPER2.2:luc* from four independent experiments).

We then evaluated circadian parameters, including period and phase offsets (Figure 4 and Figure S6). Both were estimated by fitting a damped cosine curve to the data (see Section 3). The phase offset, measured in radians, can be interpreted as the portion of a cycle between the time of synchronization (end of dexamethasone pulse) and the time of the first peak. Time-series were labeled as outliers if the period or phase offset values were more than two standard deviations away from the mean calculated across all of the replicates for a given reporter. Excluding outliers (one replicate from *mPer2:luc* and two replicates from the *hPER2.2:luc* time-series), we found the period of *hPER2.1* to be slightly longer than that of *mPer2*, while the period of *hPER2.2* was similar to *mPer2*. The average period of *mPer2* was determined to be 23.68 ± 0.72 h, which aligned with *mPer2* period values previously calculated in U2OS cells [25,26,31,43]. The average periods of *hPER2.1* and *hPER2.2* were determined to be 24.22 ± 0.51 h and 23.66 ± 0.13 h, respectively (Figure 4 and Figure S6A,B). We also determined periods by estimating the average differences in timing of the first four peaks starting 24 h after the end of the dexamethasone treatment. In these instances, the periods were found to be 23.83 ± 0.89 h, 24.65 ± 0.57 h, and 23.75 ± 0.15 h for *mPer2*, *hPER2.1*, and *hPER2.2*, respectively (Figure S6C).

The phase offset values for *mPer2* and *hPER2.2* were found to be similar (0.16π ± 0.07π rad and 0.17π ± 0.09π rad, respectively), while we observed a phase delay for *hPER2.1* (Figure 4; phase offset = 0.41π ± 0.15π rad). These results align with observations previously reported using *mPer2* promoter sequences [44]. We note that in one experiment presented here, *mPer2* periods were shorter but their phase offsets had little effect on the mean of standard deviation of the distribution. Excluding this “short-period” experiment led to an *mPer2* period of 24.2 ± 0.15 h and a phase offset of 0.12π ± 0.04π rad. We also determined the phases by calculating the difference between the first peak and its ideal timing, given a peak of bioluminescence at t = 0 h and the period estimated with damped cosine-fitting (Figure S6D). Including all experiments, those phase estimates were 2.00 ± 0.85 h for *mPer2*, 4.35 ± 1.92 h for *hPER2.1*, and 1.47 ± 1.07 h for *hPER2.2*. Excluding the short *mPer2*-period experiment, the alternate phase estimate for *mPer2* was 1.62 ± 0.77 h. Our phase offset estimates capture the phase at the end of the synchronization treatment, so period length alone does not explain the differences. In fact, the short-period *mPer2* time-series were not phase-advanced in comparison to the remaining *mPer2* time-series. *hPER2.1* time-series exhibited both delays and longer periods.

While the period and phase offsets showed variations across experiments, the values within each experiment were tightly grouped. The similarities in the period and phases of *mPer2* and *hPER2.2* can be attributed to the conserved “rhythm-generating element” located upstream of +1_m_ [21]. We hypothesize that the presence of regulatory elements in the upstream region of *hPER2.1*, in addition to the rhythm-generating element, could be responsible for the longer periods and phase delays.

## 3. Materials and Methods

### 3.1. Plasmid Construction

The *mPer2:luc* reporter construct (pMA3160 lentiviral backbone) was previously generated by our group [10]. The *hPER2.1:luc* lentiviral reporter construct containing a 781 bp truncated human *PER2* promoter fragment (−1114 to −333; relative to +1_h_) was obtained from Dr. Benedetto Grimaldi (Istituto Italiano di Tecnologia; Genova, Italy) [38]. For generating the *hPER2.2:luc* promoter reporter plasmid, a 941 bp truncated human *PER2* promoter fragment (−752 to +101, relative to +1_h_) was PCR-amplified from a pGL4[Luc2P/Neo] backbone obtained from Dr. Helmut Zarbl (Rutgers University; New Brunswick, NJ, USA) [39]. The primers used for PCR were: forward primer (containing EcoRI restriction site, underlined) = 5′-ccg gaa ttc AGG TGG AGG TCT CCC TCG TCC GGC T-3′; reverse primer (containing NotI restriction site, underlined) = 5′-AAA TAT gcg gcc gcG GAG GGT TCC CAA AAG AGA A-3′. The EcoRI/NotI containing *hPER2.2* promoter fragment was subcloned into a pMA3160 lentiviral vector (Addgene plasmid #35043, deposited by Dr. Mikhail Alexeyev) [45], thereby generating the *hPER2.2:luc* promoter reporter construct. The ligated plasmid DNA was transformed into the electrocompetent Stbl3 strain of *E. coli* (Thermo Fisher, Waltham, MA, USA), plated on LB agar plates containing ampicillin, and incubated for approximately 16–20 h at 37 °C. The recombinant clones were further expanded, and plasmid DNA was isolated using a GeneJET plasmid midiprep kit (Thermo Fisher Scientific, #K0481). The reporter plasmid was validated using Sanger sequencing and whole plasmid sequencing (Azenta Life Sciences, South Plainfield, NJ, USA).

### 3.2. Cell Culture

HEK293T (ATCC) cells were maintained in Dulbecco’s Modified Eagle Medium/Nutrient Mixture F12 (DMEM/F12; Gibco, Waltham, MA, USA) containing 10% fetal bovine serum (FBS; Corning, Corning, NY, USA) and 100 U/mL penicillin-streptomycin (Gibco). U2OS (ATCC) cells and derived reporter cell lines were cultured in Dulbecco’s Modified Eagle Medium (DMEM; Gibco) supplemented with 10% FBS, 100 U/mL penicillin-streptomycin (Gibco), 2mM glutamine (Gibco), 1mM sodium pyruvate (Gibco), and 1% non-essential amino acids (Cytiva, Marlborough, MA, USA). The cells were grown in an incubator maintained at 37 °C under 5% CO_2_ atmosphere.

### 3.3. Lentiviral Transductions

The generation of the U2OS-*mPer2:luc* cell line has been previously described [26]. For U2OS-*hPER2.1:luc* and -*hPER2.2:luc* cells, the procedure was as follows. HEK293T cells were used as the viral packaging cell line and seeded into 60 mm dishes at a density of 2.5 × 10^6^ cells per dish. At 70–90% confluence (approximately 24 h), the cell culture media was replaced with fresh HEK293T culture media. The cells were further treated with a transfection mixture containing 5 μg of the target DNA (*hPER2.1:luc*, or *hPER2.2:luc*), 3 μg of lentiviral packaging plasmid (psPAX2; Addgene plasmid #12260, deposited by Dr. Didier Trono), and 2 μg of lentiviral envelope plasmid (pMD2.G; Addgene plasmid #12259, deposited by Dr. Didier Trono), along with Lipofectamine 3000 (Invitrogen, Waltham, MA, USA), following the manufacturer’s protocol. Meanwhile, the target cell line, U2OS, was seeded into T25 flasks at a density of 6 × 10^5^ cells/flask and grown to 60–70% confluence.

After 48 h incubation, the viral supernatant from HEK293T cells was collected and sterile-filtered using a 0.45 μm syringe filter before combining it with U2OS culture media containing 4 μg/mL polybrene (Sigma-Aldrich, St. Louis, MO, USA) in a 1:1 ratio. Culture media from the U2OS cells was replaced with 6 mL of viral media, and the infections were repeated every 12 h over two days. The viral media was replaced with fresh media 24 h following the final infection. U2OS-*hPER2.1:luc* cells were passaged and screened for copGFP using flow cytometry (BD FACSAria Fusion at the Flow Cytometry Core Facility, Institute for Applied Life Sciences, UMass Amherst) to select for stably transfected cells, which were further cultured for future use. U2OS-*hPER2.2:luc* cells were treated with U2OS media containing 4 μg/mL of puromycin (Gibco) for 3–5 weeks, before expanding and freezing for future use.

### 3.4. Cell Synchronization and Bioluminescence Recording

For luminometry experiments, the U2OS-*mPer2:luc*, -*hPER2.1:luc*, and -*hPER2.2:luc* cells were seeded in 35 mm dishes at a density of 4 × 10^5^ cells per dish. After approximately 24 h, the cells were rinsed with PBS and replaced with synchronization media containing 100 nM dexamethasone (Sigma-Aldrich) in U2OS cell culture media for two hours. After synchronization, the cells were rinsed with phosphate-buffered saline (PBS) and treated with recording media. Recording media was prepared by dissolving powdered DMEM (Sigma-Aldrich) in water (11.25 mg/mL). The resulting solution was sterile-filtered using a 0.22 μm syringe filter and further supplemented with 5% FBS, 10 mM HEPES (Cytiva), 4 mM sodium bicarbonate (Fisher Bioreagents, Waltham, MA, USA), 500 U/mL penicillin-streptomycin, and 500 μM D-luciferin (Thermo Scientific) dissolved in water. After the addition of recording media, the dishes were sealed with 40 mm cover glasses using autoclaved silicone vacuum grease, and the bioluminescence recordings were monitored using a Lumicycle 32 system (Actimetrics, Wilmette, IL, USA) for 7 days at 37 °C.

### 3.5. Data Analysis

The bioluminescence recordings were preprocessed to exclude the first 24 h of transient expression and discard the oscillations after 6 days. The data was then detrended by removing a 24 h sliding window and fit into a damped cosine curve with a linear baseline $Ae^{-\lambda t}\cos\left(\frac{2\pi }{\tau }-\theta \right)+c_{o}+c_{1}t$, where *τ* is the period in hours, *θ* is the phase in radians, and *t* is the time in hours. The bioluminescence time series was considered an outlier if the goodness-of-fit value was less than 0.8, or if the period and phase offset values were greater than two standard deviations away from the mean for all the replicates, for a given reporter.

## 4. Conclusions

PER2 is an integral component of the mammalian core clock. Prior studies have shown that the negative feedback established by PER2 governs the length of the circadian period [44,46]. The murine *Per2* (*mPer2*) promoter has been studied extensively to recognize important regulatory elements that are responsible for the rhythmic behavior of the mammalian clock. Luciferase-based reporters driven by truncated sections of *mPer2* promoters have been used to understand the fundamental circadian behavior of *Per2* in homeostasis and disease. However, most of the reporters developed to date have utilized murine sequences in in vitro cell lines of both murine and human origin. While known, important regulatory elements are conserved across species, we should not disregard any possible variations and their effects arising from the unconserved sections of the *Per2* gene.

In this work, we aimed to minimize species-specific variability by developing luciferase reporters driven by a human-derived *PER2* promoter for use in in vitro cell lines of human origin. We utilized lentiviral luciferase constructs with two different sections of the *hPER2* promoter (*hPER2.1* and *hPER2.2*). Both promoters had a region of overlap, with *hPER2.1* aligning with the upstream region of the endogenous promoter, while *hPER2.2* aligned with the downstream region. Comparing the oscillations of both *hPER2:luc* reporters with murine *Per2:luc* in the commonly employed U2OS circadian model, we found that the human-derived promoters exhibited robust oscillations. Interestingly, we also noted that the upstream frame of the *hPER2* promoter triggered a phase delay and a slightly longer period. This finding corroborated previous observations in *mPer2* promoter sequences, highlighting the presence of phase-delaying elements in addition to the rhythm-generating element.

A possible explanation for the higher amplitudes in *hPER2:luc* cell lines is the use of longer promoter sequence frames. The *mPer2:luc* reporter used was designed to include the minimal region of the *Per2* promoter that establishes circadian gene expression [21]. For *mPer2*, it has been shown that promoter lengths can affect amplitudes and that increasing them upstream of the E’-box can result in phase delays [22]. The use of longer frames for the *hPER2:luc* reporters could allow for synergistic cooperation between neighboring elements, resulting in a more dynamic expression pattern. While more research needs to be conducted to understand the regulatory mechanisms responsible for the variations in amplitudes and phases in human-derived clock components, our work provides a solid foundation and introduces reliable human-derived reporter sequences to track *hPER2* transcription. In the future, these *hPER2:luc* sequences should be used to investigate circadian changes within the human core clock and to better understand the fundamental aspects of the negative feedback regulation in both health and disease.

## Data Availability

The original data presented in this study are openly available via the Data Repository at ScholarWorks. The URL is https://hdl.handle.net/20.500.14394/57756 (accessed on 24 September 2025).

## Supplementary Materials

The following supporting information can be downloaded at: https://www.mdpi.com/article/10.3390/ijms262110785/s1.

## Abbreviations

The following abbreviations are used in this manuscript:

SCN	Suprachiasmatic nucleus
TTFL	Transcriptional-translational feedback loop
BMAL1	Brain and muscle Arnt-like-1
CLOCK	Circadian locomotor output cycles kaput
PER	Period
CRY	Cryptochrome
ChIP	Chromatin immunoprecipitation
RT-PCR	Reverse transcription-polymerase chain reaction
T-Coffee	Tree-based consistency objective function for alignment evaluation
MSA	Multiple sequence alignment
HRE	Hypoxia response element
luc	Luciferase
TSS	Transcription start site
PCR	Polymerase chain reaction
LB	Luria broth
DMEM	Dulbecco’s modified eagle medium
FBS	Fetal bovine serum
PBS	Phosphate-buffered saline
