# Supplementary material for: Promoter Frame Position Affects Strength and Nature of Circadian Oscillations in hPER2 Luciferase Reporters
Source: Int J Mol Sci. 2025 Nov 6;26(21):10785. doi: 10.3390/ijms262110785 (PMC12608312; doi:10.3390/ijms262110785)
Supplement: Supplementary file 1 [file ijms-26-10785-s001.zip › ijms-3925978-supplementary.pdf]

Supplementary Materials

Promoter Frame Position Affects Strength and Nature of Circadian Oscillations in *hPER2* Luciferase Reporters

Bhavna Kalyanaraman [1], Gabrielle Villafana [2], Stephanie R. Taylor [3], and Michelle E. Farkas [1,2] *

[1] Department of Chemistry, University of Massachusetts Amherst, Amherst, MA 01003, USA

[2] Molecular and Cellular Biology Graduate Program, University of Massachusetts Amherst, Amherst, MA 01003, USA

[3] Department of Computer Science, Colby College, Waterville, ME 04901, USA; srtaylor@colby.edu

* Correspondence: farkas@chem.umass.edu

Luciferase Assay

U2OS-*mPer2:luc*, U2OS-*hPER2.1:luc*, and U2OS-*hPER2.2:luc* cells were plated in white-walled 96-well plates at a cell seeding density of 1 x 10^4 cells per well. U2OS (non-manipulated) cells were used as a blank control. At confluence, the cells were rinsed with phosphate-buffered saline (PBS; Gibco) and replaced with fresh U2OS cell culture medium and ONE-Glo EX luciferase assay reagent (Promega #N1610). The cells were incubated for 5 minutes, and the bioluminescence (550 nm) was recorded using a SpectraMax iD3 multi-mode microplate reader.

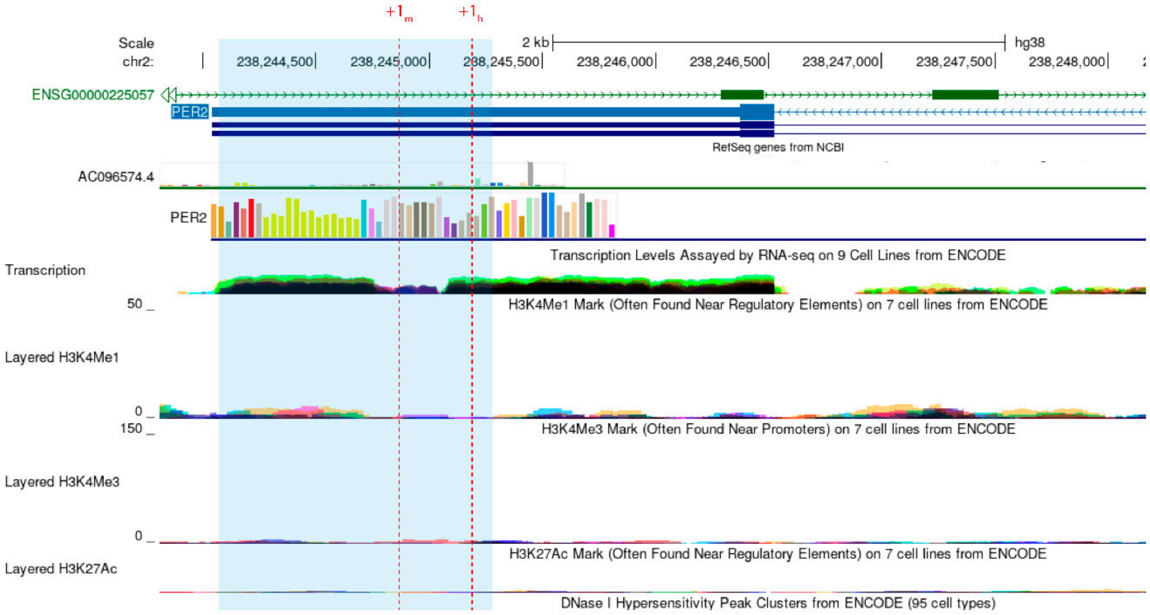

Figure S1. ENCODE data for the hPER2 gene. The full-length hPER2 promoter (-1121 to +101) is highlighted in blue. The $+1_m$ and $+1_h$ sites are indicated using dashed lines (in red). H3K4Me1 and H3K27Ac tracks indicate enhancer/regulatory regions on the hPER2 gene. H3K4Me3 activity is enriched in active promoters and indicates the presence of transcriptional initiation complexes.

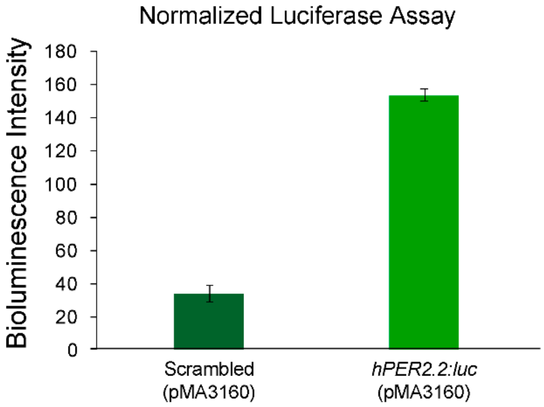

Figure S2. Luciferase assay to assess the activity of the *hPER2.2:luc* promoter-reporter. The luminescence output for *hPER2.2:luc* was compared against that from a control containing the same lentiviral backbone, with a non-specific (scrambled) insert. The data shown for each construct is an average of three biological replicates (N=3). The luminescence intensities have been normalized to the baseline luminescence from non-transfected U2OS cells.

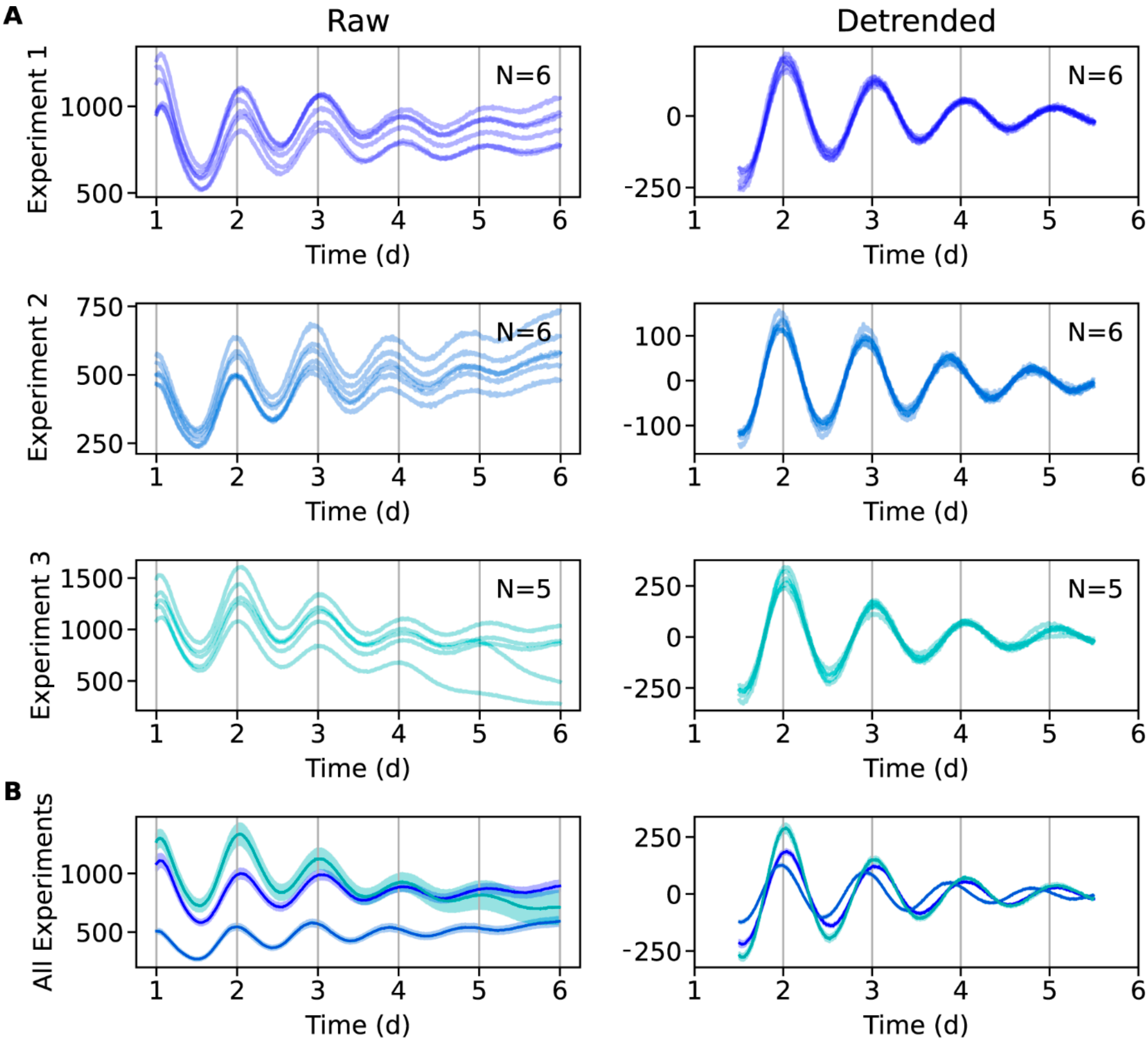

Figure S3. Bioluminescence time-series for *mPer2:luc* for three experiments. (A) Shown are the time-series for each experiment. Excluding a 24-h transient, individual time-series are shown in raw (left) and de-trended (the average of a 24-h moving window subtracted from every point; right) forms. (B) The mean (raw or de-trended) of all time-series for each experiment is plotted as a solid line, with the standard error of the mean as a semi-transparent envelope around it. Colors correspond to experiment.

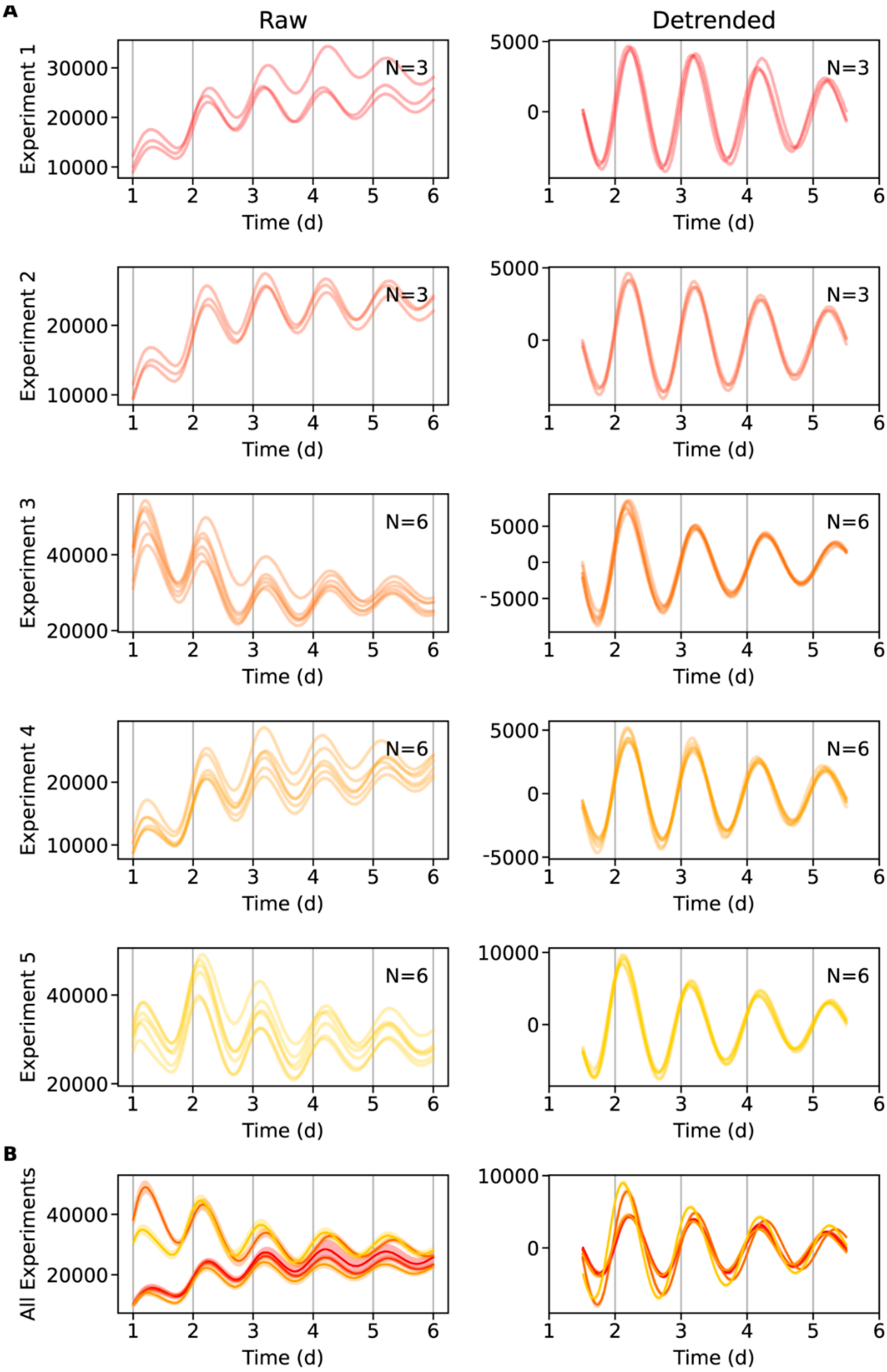

Figure S4. Bioluminescence time-series for *hPER2.1:luc* for five experiments. (A) Shown are the time-series for each experiment. Excluding a 24-h transient, individual time-series are shown in raw (left) and de-trended (the average of a 24-h moving window subtracted from every point; right) forms. (B) The mean (raw or de-trended) of all time-series for each experiment is plotted as a solid line, with the standard error of the mean as a semi-transparent envelope around it. Colors correspond to experiment.

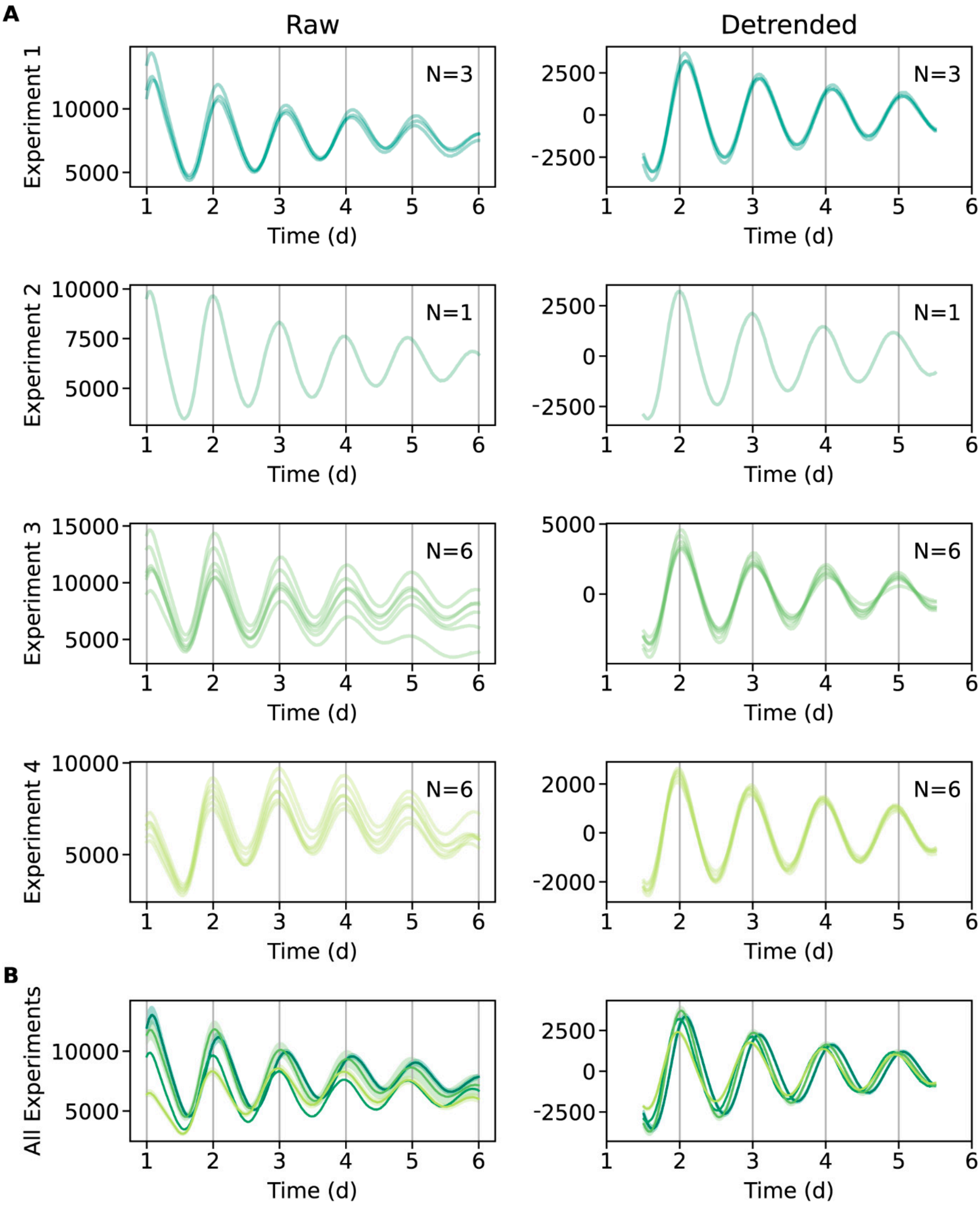

Figure S5. Bioluminescence time-series for *hPER2.2:luc* for four experiments. (A) Shown are the time-series for each experiment. Excluding a 24-h transient, individual time-series are shown raw (left) and de-trended (the average of a 24-h moving window subtracted from every point; right). (B) The mean (raw or de-trended) of all time-series for each experiment is plotted as a solid line, with the standard error of the mean as a semi-transparent envelope around it. Colors correspond to experiment.

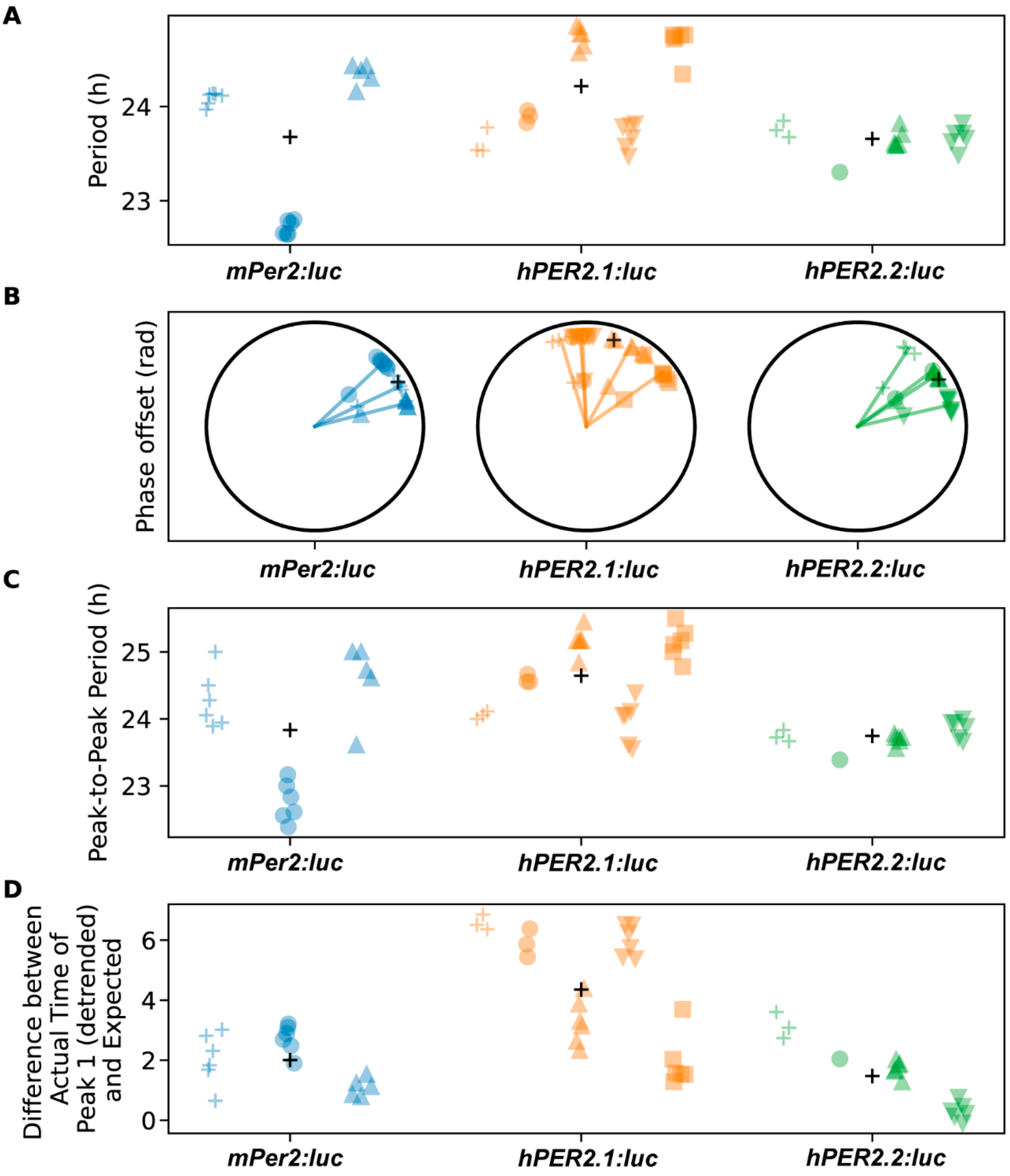

Figure S6. Shown are the periods and phase offsets of *mPer2:luc*, *hPER2.1:luc*, and *hPER2.2:luc* time-series, separated by experiment. Shown are the period (A) and phase-offset (B) of the best-fit damped cosine curve as shown in the main text, the period as estimated by the average difference in timing of the first four peaks starting 24 hours after the end of Dex treatment (C), and a phase offset calculated as the actual time of the first peak minus the predicted time, assuming the rhythm begins with a peak at time t=0 using the period estimated from fitting the damped cosine curve (D). For A, C, and D, the averages across all experiments are indicated by a black plus sign (+). For B, the average for each experiment is indicated by the sign for that experiment marked mid-way through the line. For each parameter and each promoter, each experiment is indicated by the following markers: pluses (+) for Experiment 1, circles (●) for Experiment 2, triangles (▲) for Experiment 3, inverted triangles for Experiment 4 (▼; where applicable), and squares for Experiment 5 (■; where applicable). For the jitter plots (A, C, D), the experiment is also indicated by position. Experiments are shown in the same order as in Figures S3, S4, and S5.
